# HLA Associations and Clinical Implications in T-Cell Mediated Drug Hypersensitivity Reactions: An Updated Review

**DOI:** 10.1155/2014/565320

**Published:** 2014-05-08

**Authors:** Chi-Yuan Cheng, Shih-Chi Su, Chi-Hua Chen, Wei-Li Chen, Shin-Tarng Deng, Wen-Hung Chung

**Affiliations:** ^1^Department of Pharmacy Administration, Chang Gung Memorial Hospital, Linkou 33305, Taiwan; ^2^Department of Dermatology, Drug Hypersensitivity Clinical and Research Center, Chang Gung Memorial Hospitals, Linkou 33305, Taiwan; ^3^College of Medicine, Chang Gung University, Taoyuan 33302, Taiwan; ^4^Department of Dermatology, Keelung Chang Gung Memorial Hospital, No. 222 Maijin Road, Anle District, Keelung City 20448, Taiwan

## Abstract

T-cell mediated drug hypersensitivity reactions may range from mild rash to severe fatal reactions. Among them, drug reaction with eosinophilia and systemic symptoms (DRESS) or drug-induced hypersensitivity syndrome (DIHS), Stevens-Johnson syndrome/ toxic epidermal necrolysis (SJS/TEN), are some of the most life-threatening severe cutaneous adverse reactions (SCARs). Recent advances in pharmacogenetic studies show strong genetic associations between human leukocyte antigen (HLA) alleles and susceptibility to drug hypersensitivity. This review summarizes the literature on recent progresses in pharmacogenetic studies and clinical application of pharmacogenetic screening based on associations between SCARs and specific HLA alleles to avoid serious conditions associated with drug hypersensitivity.

## 1. Introduction

Severe cutaneous adverse reactions to drugs (SCARs) include syndromes such as Stevens-Johnson syndrome/toxic epidermal necrolysis (SJS/TEN) and drug reaction with eosinophilia and systemic symptoms (DRESS) or drug-induced hypersensitivity syndrome (DIHS) [1, 2]. Although the incidence of SJS and TEN is very low (0.4–6 cases per million persons per year), they are severe, life-threatening adverse drug reactions with mortality rates as high as 5% to 12.5% for SJS and 50% for TEN [1, 3]. In patients with DRESS, skin rash is usually associated with fever, hepatitis and/or other internal organ involvement, lymphadenopathy, and hematological abnormalities (leukocytosis, eosinophilia, and atypical lymphocytosis). DRESS is also a rare adverse drug reaction and often has a relapsing-remitting course despite withdrawal of the drug. Reactivation of human herpesvirus (HHV), mainly HHV-6 and less frequently cytomegalovirus, has been described during the course of DRESS [4–7]. These viral reactivations have been reported in association with recurrence of symptoms more than 2 weeks after the drug was discontinued [5, 6]. SCARs are generally considered unpredictable irrespective of dosage.

In this decade, an important progress for the underlying mechanisms of SCARs has been achieved by the discovery of association between HLA alleles and SCARs. In 2004, we first reported a strong association of HLA-B^∗^15:02 with carbamazepine-induced SJS/TEN [8]. More importantly, we found that HLA-B^∗^15:02 is specific for the carbamazepine-induced activation of cytotoxic T lymphocytes (CTLs) in SJS/TEN patients. Upon the stimulation, CTLs expressed a large amount of granulysin which was identified as a key mediator responsible for the extensive epidermal necrolysis in SJS/TEN [9].

In this review, we summarize recent progresses in identifying genetic links for SCARs or drug-induced hypersensitivity reactions, related to carbamazepine and other aromatic anticonvulsants (phenytoin, oxcarbazepine, and lamotrigine), allopurinol, antiretroviral drugs (abacavir, nevirapine), and dapsone. Reported findings were summarized and listed in Table 1.

## 2. Pharmacogenetics of SCARs and Delayed-Type Drug Hypersensitivity

### 2.1. HLA Alleles Associated Drug Hypersensitivity

#### 2.1.1. Carbamazepine and Other Aromatic Amine Anticonvulsants

*(1) Carbamazepine.* Carbamazepine (CBZ), an aromatic amine anticonvulsant, is approved by the US Food and Drug Administration for the treatment of epilepsy and other seizure disorders, trigeminal neuralgia, and bipolar disorder. Approximately 10% of patients develop mild cutaneous adverse reactions, for example, maculopapular eruption (MPE), within the first 3 months of CBZ therapy [10], and the incidence of CBZ-induced SJS/TEN is very low (incidence 0.23% in Taiwan) [11]. In addition, long-term use of CBZ may increase risk of lupus, mostly in women [12].

*Phenotype and Ethnicity Specific Association with Specific HLA Alleles of CBZ-Induced SCARs*. The most striking evidence of genetic susceptibility to SJS/TEN was provided by our findings that HLA-B^∗^15:02 is strongly associated with CBZ-induced SJS/TEN [8]. We also found that the HLA association is phenotype-specific in CBZ-induced cutaneous reactions in Taiwan, as HLA-B^∗^15:02 is only associated with SJS/TEN, and HLA-A^∗^31:01 is only associated with MPE and DRESS [13]. A following study in Hong Kong Han Chinese also found that the presence of HLA-B^∗^15:02 greatly increased the risk of CBZ-induced SJS/TEN but not MPE or DRESS [14]. The association of CBZ-induced SJS/TEN and HLA-B^∗^15:02 was subsequently reported in China, as well as in other South/Southeast Asian areas including Thailand, Malaysia, Indian, Vietnam, Cambodia, and Reunion Islands [15]. Recently, systematic reviews and meta-analyses verified strong relationship between the HLA-B^∗^15:02 allele and CBZ-induced SJS/TEN in Han Chinese and certain Southeast Asian populations [16, 17]. However, the HLA-B^∗^15:02 allele has not been observed in cases of CBZ-induced SJS/TEN in other ancestral groups such as Japanese and Korean populations or non-Asian descendants in Europe or North America [15]. These observations may be explained by the various HLA allele frequencies in the different populations in the world. The allele frequency of HLA-B^∗^15:02 among residents of Southeast Asian countries was reported to be 1–8%; however, it is absent or lower than 0.4% in Europeans or Northeast Asians (e.g., Japan and Korea) [15]. By comparison, Ozeki et al. and McCormack et al. showed that HLA-A^∗^31:01 is the main genetic determinant for all types of CBZ-induced adverse cutaneous reactions, including SJS, TEN, MPE, and DRESS in the Japanese and Europeans [18, 19]. However, there were only very few cases of CBZ-induced SJS/TEN which are included in these analyses. Recently, a follow-up study with a larger sample size from Taiwan and RegiSCAR showed that HLA-A^∗^31:01 was strongly associated with CBZ-induced DRESS in the Europeans (OR 57.6; 95% CI 11.0–340; *P* < 0.001) as well as in Han Chinese (OR 23.0; 95% CI 4.2–125; *P* < 0.001), but HLA-A^∗^31:01 had no association with CBZ-induced SJS/TEN in Han Chinese and the Europeans [20]. A meta-analysis confirmed that in all populations, HLA-A^∗^31:01 had a strong association with CBZ-induced DRESS (OR 13.2; 95% CI 8.4–20.8; *P* < 0.001), but a much weaker association with CBZ-induced SJS/TEN (OR 3.94; 95% CI 1.4–11.5; *P* = 0.01) [20]. This finding suggested that HLA-A^∗^31:01 may not be a clinically useful marker for preventing life-threatening SJS/TEN induced by CBZ.

Furthermore, our more recent study with increased sample size (112 CBZ-SJS/TEN) showed that the strength of HLA-B^∗^15:02 association with CBZ-induced SJS/TEN was also related to the clinical severity. CBZ-SJS/TEN showed the strongest association (100%, 25/25) with the HLA-B^∗^15:02 allele in SJS/TEN patients if they had more than 5% body surface area (BSA) skin detachment but lost its 100% association (85.1%, 74/87) in SJS with <5% BSA detachment [21].

*Other HLA Alleles Link to CBZ-Induced SJS/TEN*. Some of the cases of CBZ-induced SJS/TEN did not carry the HLA-B^∗^15:02 allele, suggesting that other genetic variants may play a role. HLA-B^∗^15:02 belongs to HLA-B75. Other members of HLA-B75 serotype have been implicated in CBZ-induced SJS/TEN, such as HLA-B^∗^15:08 in the Indians [15], HLA-B^∗^15:11 in the Koreans [22], the Japanese [23], and Han Chinese in China [24], and Taiwan (our unpublished data), and HLA-B^∗^15:18 in Japanese [25]. The rationale is that these HLA alleles share high amino acid sequence homology that may resemble structural features of HLA-B^∗^15:02 and thus may be able to trigger a similar cutaneous adverse reaction to CBZ [26].

Several studies have suggested that certain HLA allele may exert a protective effect against CBZ-induced SJS/TEN, as evidence by lower carrier frequencies in cases compared with CBZ-tolerant controls, including HLA-B^∗^40:01 in Taiwan [13] and Hong Kong [27] and HLA-B^∗^07:02 in the Caucasians [28]. However, whether these alleles have functional roles for the protective effect needed to be further studied.

Clinical Implications of HLA-B*15:02 Finding for CBZ-SJS/TEN. Screening for HLA-B^∗^15:02 has been recommended by the US Food and Drug Administration prior to starting CBZ in patients with Asian ancestry, particularly for those of Southeast Asian ancestry since December 2007. Based on Clinical Pharmacogenetics Implementation Consortium (CPIC) guidelines for carriers of HLA-B^∗^15:02, if the patient is CBZ-naïve, no use of CBZ is strongly recommended regardless of ancestry, and if the patient has previously used CBZ for longer than 3 months without incidence of cutaneous adverse reactions, use of CBZ is cautiously considered [10].

The translation of HLA-B^∗^1502 and CBZ-induced SJS/TEN from discovery to a guideline-based test used routinely in Taiwan is a notable example (Table 2) [29]. Since 2010 in Taiwan, the National Health Insurance has covered the expense of the genetic screening for HLA-B^∗^15:02 in individuals initiating CBZ. The success of HLA-B^∗^15:02 screening in reducing the incidence of CBZ-induced SJS/TEN has been demonstrated clinically from 23 hospitals in Taiwan [11]. After applying genetic screening for HLA-B^∗^15:02 prior to initiating CBZ, there was no cases of either SJS or TEN reported. This outcome was significant since approximately 8 cases were expected based on the estimated historical incidence of 0.23% for CBZ-induced SJS/TEN in Taiwan [11].

*(2) Other Aromatic Amine Anticonvulsants (Phenytoin, Oxcarbazepine, and Lamotrigine)*. Phenytoin, oxcarbazepine, lamotrigine, and CBZ are metabolized to arene oxide metabolites. Our study revealed that HLA-B^∗^15:02 was associated with an increased risk of SJS/TEN upon exposure to phenytoin, oxcarbazepine, and potentially lamotrigine in a Taiwanese population although the strength of these associations was weaker than that of CBZ [30]. Small case studies in Thailand (4 cases phenytoin induced SJS) and Hong Kong (single cases of phenytoin and lamotrigine induced SJS) also showed the presence of HLA-B^∗^15:02 in all SJS patients [14, 31]. Recently, a meta-analysis of the relationship between aromatic amine anticonvulsants-induced SJS/TEN and HLA-B^∗^15:02 in Han Chinese populations showed a strong association of HLA-B^∗^15:02 with phenytoin (OR 4.26; 95% CI 1.93–9.93; *P* < 3 × 10^−4^) and, to a lesser extent, with lamotrigine (OR 3.59; 95% CI 1.15–11.22; *P* = 0.03) [27]. These studies confirmed a clinically relevant association between the HLA-B^∗^15:02 allele and phenytoin-induced SJS/TEN, supporting the US Food and Drug Administration recommendation that health care providers should consider avoiding phenytoin and its prodrug, fosphenytoin, as alternatives for CBZ in HLA-B^∗^15:02 carriers (http://www.fda.gov/Drugs/DrugSafety/PostmarketDrugSafetyInformationforPatientsandProviders/ucm110259.htm).

#### 2.1.2. Allopurinol

Allopurinol is a drug that has been used for decades to lower the levels of serum urate in patients with gout and in cancer patients undergoing chemotherapy at risk of tumor lysis syndrome. The greatest safety concern with allopurinol is an estimated 0.1–0.4% risk of SCARs [32]. Allopurinol is a major cause of SCARs, and one of the most serious, with up to 25% mortality [32]. The association of HLA-B^∗^58:01 with allopurinol-induced SCARs was first identified in Taiwan (our published data) [32]. In our study, HLA-B^∗^58:01 was present in 100% (51/51) of the patients with allopurinol-induced SCARs, as compared with 15% (20/153) of allopurinol-tolerant controls and 20% (19/93) of the population controls [33]. This strong association was replicated in Han Chinese in China [32] and Thai populations [32]. A similar but more modest association was observed in Korean, Japanese, and European populations [32]. One systematic review and meta-analysis that consolidated the published studies showed a strong and significant association between HLA-B^∗^58:01 and allopurinol-induced SJS/TEN with odds ratio of 74.18 (95% CI 26.95–204.14) and 101.45 (95% CI 44.98–228.82) for Asian and non-Asian populations, respectively [34]. Given the high specificity for allopurinol-induced SCARs, allopurinol is contraindicated in patients who have tested positive for HLA-B^∗^58:01 according to the CPIC guidelines for HLA-B genotype and allopurinol dosing [32].

The prospective studies of HLA-B^∗^58:01 genotyping for the prevention of allopurinol-induced SCARs have not been published; however, one such study is under way in Taiwan [35]. A cost-effectiveness analysis for prevention of allopurinol-induced adverse drug reaction based on a retrospective study of Taiwan National Health Insurance database from year 2002 to 2011 revealed that the genetic test for HLA-B^∗^58:01 in high-risk population, defined as patients older than 60 years or patients with renal dysfunction, was expected to recoup the investment in an average of 8.6 years, and the alternative treatment with febuxostat takes only 1.1 years (our unpublished data). The Taiwan Department of Health has updated the labeling for allopurinol to include information on HLA-B^∗^58:01 (http://www.tdrf.org.tw/ch/03_message/mes_02_main.asp?bull_id=3529), although the US Food and Drug Administration has not. The updated label describes the strong association between HLA-B^∗^58:01 and allopurinol-induced SCARs in the Han Chinese population and recommends testing for the allele before the use of allopurinol.

Based on the data from the Han Chinese and Thai populations regarding strong associations between HLA-B^∗^58:01 and allopurinol-induced SCARs, the positive predictive value for HLA-B^∗^58:01 is only 1.5% and the negative predictive value is 100% [33, 36]. Therefore, a large number of patients carrying the allele will not develop SCARs when they receive allopurinol treatment. New genetic factors may be identified in the future to differentiate the HLA-B^∗^58:01 carriers who are or are not likely to develop SCARs. However, apart from Han Chinese and Thai populations, the absence of HLA-B^∗^58:01 does not totally eliminate the possibility of developing SCARs, especially in the European population [32].

In addition to SCARs, allopurinol therapy is also associated with a 2-3% incidence of MPE, a mild cutaneous adverse reaction [32]. Strong association between HLA-B^∗^58:01 and MPE was found in mainland China (OR, 339.00, *P* < 0.0001) [37], but not in Australia [38]. However, it should be emphasized that given the small number of subjects, further studies with a larger sample size will be necessary to provide a more definite answer. Recently, American College of Rheumatology's new guidelines on gout has recommended patients of Asian backgrounds to consider genetic test for HLA-B^∗^58:01 before use of allopurinol [39].

Alternative therapies in the setting of gout now are available. Febuxostat was approved by the US Food and Drug Administration in 2009. It is a nonpurine xanthine oxidase inhibitor that is primarily metabolized in the liver to inactive glucuronide and excreted into the urine and bile. Therefore, febuxostat can be used in patients with mild-to-moderate renal dysfunction without dosage adjustment. Moreover, it was reported to be tolerated in patients with a history of severe allopurinol hypersensitivity [40]. The launch of febuxostat is a milestone in the treatment of gout, while an alternative is pegloticase. In 2010, the US Food and Drug Administration approved pegloticase as an orphan drug for treating refractory chronic gout patients who were irresponsive or intolerant to other urate-lowering drugs [41]. Patients with chronic kidney disease and positive for HLA-B^∗^58:01 have been shown to be at higher risk of allopurinol-induced SCARs [42]. Hence, choosing those new drugs (e.g., febuxostat) instead of allopurinol is safer in this group of patients.

#### 2.1.3. Abacavir

Abacavir belongs to the family of nucleoside reverse transcriptase inhibitors used for the treatment of HIV-1 infection. Abacavir hypersensitivity syndrome (ABC HSS) differs from DRESS induced by other drugs, occurring at a median value of 9 days after first initiation of the drug [43]. Unlike DRESS associated with other drugs, eosinophilia and hepatitis are uncommon in patients with ABC HSS. Symptoms of ABC HSS are nonspecific and include fever, malaise, gastrointestinal symptoms, and internal organ involvement. Rash tends to be mild-to-moderate, occurring in 70% of patients with ABC HSS and often late in the course of disease. The characteristic of the ABC HSS is that symptoms will completely subside within 3 days of discontinuation [44]. Although abacavir has been commonly associated with a drug hypersensitivity syndrome in 8% of patients who start the drug [43], there have been only two published case reports of SJS/TEN potentially associated with abacavir in the literature despite over 10 years of postmarketing experience [45, 46].

In 2002, two groups independently published a strong association between HLA-B^∗^57:01 and abacavir hypersensitivity in Australian and British populations [47, 48]. Abacavir-induced hypersensitivity is present at a high frequency only in Caucasians and at a very low frequency in Asian and Black populations [49, 50]. Because of a considerable rate of false-positive result of HLA-B^∗^57:01 test, abacavir patch testing was used as a specific test to identify true abacavir hypersensitivity [51–54].

#### 2.1.4. Nevirapine

Nevirapine is a nonnucleoside reverse transcriptase inhibitor, widely prescribed for HIV-1 infection. DRESS occurs in approximately 5% of those starting nevirapine and typically associated with fever, rash, and hepatitis and SJS/TEN in 0.3% or less [55].

Different Class I and Class II HLA associations with nevirapine-induced rash and DRESS across different populations have been described. A population-based study from Western Australia revealed an association of the Class II allele HLA-DRB1^∗^01:01 with rash-associated hepatitis in those with a CD4+ ≧25% [56]. A study in Thailand showed a high frequency of HLA-B^∗^35:05 (17.5%) in patients with nevirapine-induced rash and DRESS [57]. HLA-Cw8 and HLA-B^∗^14:02 associations with nevirapine-induced DRESS were also reported in a Sardinian population [58], and a HLA-Cw8 association was noted in a Japanese population [59]. To date, no specific HLA association has been described in nevirapine-induced SJS/TEN. Recently, Ciccacci et al. suggested that genetic variability in a nevirapine-metabolizing cytochrome P450 2B6 can contribute to the susceptibility to nevirapine-induced SJS/TEN in an Italian study [60].

#### 2.1.5. Dapsone

Dapsone is used in the treatment of infections (e.g., leprosy) and chronic inflammatory diseases characterized by the infiltration of neutrophils or eosinophils (e.g., dermatitis herpetiformis). The dapsone-induced DRESS develops in 0.5 to 3.6% of persons treated with the drug [59]. Recently, it is reported that HLA-B^∗^13:01 was associated with dapsone-induced DRESS (OR 20.53; 95% CI 11.55–36.48; *P* = 6.84 × 10^−25^) among patients with leprosy in mainland China [61].

### 2.2. Genetic Associations Other Than HLA Alleles in Drug Hypersensitivity

Apart from HLA alleles association with drug hypersensitivity, contributions of genetic variants of metabolic enzymes in cutaneous adverse drug reactions had been proposed as well. Nevertheless, most of these results were either frustrating or with uncertain till now. We mention sulfonamide and phenytoin as examples. NAT2 allele encoding N-acetyltransferase 2, a major enzyme in the sulfonamide metabolism. Recently, Sacco et al. found no association of NAT2 coding alleles with sulfonamide hypersensitivity (predominantly cutaneous eruptions) [62]. Cytochrome P_450_ (CYP) 2C9, a main metabolic enzyme for phenytoin, may be the pathological mechanism for phenytoin-induced cutaneous adverse drug reactions. From a Korean study, they found that a heterozygous CYP2C9^∗^3 variant could play a role in the proportion of patients with phenytoin-induced cutaneous adverse drug reactions [63]. However, there were only very few cases included. Further investigations with a larger sample size are warranted.

## 3. Conclusions

Studies presented in this review show the tremendous progress in the area of pharmacogenetics of SCARs in recent years. In Taiwan, personalized medicine has started identifying patients at high risk of SCARs based on pharmacogenomics by using biomarkers, such as HLA-B^∗^15:02 for carbamazepine, HLA-B^∗^58:01 for allopurinol, and resulting in the reduction of serious cutaneous adverse drug reactions. However, biomarkers have only been identified for a few, such as abacavir, carbamazepine, and allopurinol. It is likely that further progress will be made by the nationwide or even international well-defined sampling of patients to identify corresponding genetic markers that can predict patients at high risk on the basis of ethnicity and the causative drug.

## Figures and Tables

**Table 1:** Associations of SCARs and delayed-type drug hypersensitivity and HLA alleles.

Causative drug	HLA allele	Hypersensitivity reactions	Ethnicity	Odds ratio (95% CI)	Reference
Abacavir	B*57:01	Abacavir hypersensitivity	Caucasians	117 (29–481)	[48]
Allopurinol	B*58:01	SJS/TEN/DRESS	Asians	74.18 (26.95–204.14)	[34]
			Non-Asians	101.45 (44.98–228.82)	[34]
Carbamazepine	B*15:02	SJS/TEN	Han Chinese	115.32 (18.17–732.13)	[17]
			Thai	54.43 (16.28–181.96)	[17]
			Malaysians	221.00 (3.85–12694.65)	[17]
			Indians	54.60 (2.25–1326.20)	[17]
	B*15:08		Indians	nd	[15]
	B*15:11		Japanese	16.3 (4.76–55.61)	[23]
			Koreans	18.0 (2.3–141.2)	[22]
			Mainland China Han Chinese	31.00 (2.74–350.50)	[24]
	B*15:18		Japanese	13.58 (nd)	[25]
	A*31:01	DRESS	Han Chinese	23.0 (4.2–125)	[20]
			Europeans	57.6 (11.0–340)	[20]
		SJS/TEN	Europeans	4.4 (1.1–17.3)	[20]
			All populations	3.94 (1.4–11.5)	[20]
		SJS/TEN	Europeans	25.93 (4.93–116.18)	[19]
		DRESS	Europeans	12.41 (1.27–121.03)	[19]
		MPE	Europeans	8.33 (3.59–19.36)	[19]
		SJS/TEN/DRESS	Japanese	10.8 (5.9–19.6)	[18]
Dapsone	B*13:01	DRESS	Mainland China Han Chinese	20.53 (11.55–36.48)	[61]
Lamotrigine	B*15:02	SJS/TEN	Han Chinese	3.59 (1.15–11.22)	[27]
Nevirapine	DRB1*01:01	DRESS/MPE	Australians	depend on CD4 T-cells count	[56]
	B*14:02		Sardinians	nd	[58]
	B*35:05		Thai	18.96 (4.87–73.44)	[57]
	Cw8		Sardinians, Japanese	nd	[58, 59]
Oxcarbazepine	B*15:02	SJS/TEN	Taiwan Han Chinese	80.7 (3.8–1714.4)	[30]
Phenytoin	B*15:02	SJS/TEN	Han Chinese	4.26 (1.93–9.39)	[27]

DRESS: drug reaction with eosinophilia and systemic symptoms.

MPE: maculopapular eruption.

SCARs: severe cutaneous adverse reactions to drugs.

SJS: Stevens-Johnson syndrome.

TEN: toxic epidermal necrolysis.

CI: confidence interval.

nd: no data.

**Table 2:** Translational roadmap of CBZ-SJS research and its clinical applications [24].

*1998–2004 *	
CBZ, an aromatic anticonvulsant, was recognized as the major cause of SJS, as listed in the records of Taiwan Drug Relief Foundation.	
*2004 *	
A strong association of CBZ-induced SJS with HLA-B∗15:02 in Han Chinese was first uncovered in Taiwan.	
*2006 *	
The correlation of CBZ-induced SJS with HLA-B∗15:02 was not existent in Caucasian patients, indicating an ethnical specificity of HLA-B∗15:02 allele.	
*2007-2008 *	
The association of CBZ-induced SJS with HLA-B∗15:02 was found among many populations in Southeast Asia.	
*2007 *	
The US Food and Drug Administration has published an alert to healthcare professionals on the use of CBZ to Asians. (The incidence rate of CBZ-induced SJS is 5.9/10000 in Taiwan and 0.2/10000 in the US.)	
*2007 *	
The Taiwan and US Food and Drug Administration relabeled the drug information of CBZ and recommended a genetic screening of HLA-B∗15:02 prior to starting CBZ in patients with Asian ancestry, particularly for those of Southeast Asian ancestry.	
*2010 *	
The National Health Insurance in Taiwan has covered the expense of the genetic screening for HLA-B*15:02 in individuals initiating CBZ.	

CBZ: carbamazepine; SJS: Stevens-Johnson syndrome; HLA: human leukocyte antigen.
